# When policy meets reality: the new 18-hour on-call shift policy and the Israeli anesthesia workforce crisis

**DOI:** 10.1186/s13584-023-00556-x

**Published:** 2023-03-02

**Authors:** Ariel Wimpfheimer, Charles Weissman, Shai Fein, Yehuda Ginosar, The Israel Anesthesiology Workforce Study Group, Haled Abd-Al-Halim, Hakeem Abu-Rais, Chaim Berkenstadt, Ilya Chernoy, Maruan Armaly, Yaakov Duvdivani, Leonid Eidelman, Shai Fine, Brian Fredman, Yulia Gadulov, Zeev Goldik, Yaakov Gozal, Zoya Haituv, Alex Izakson, Yaakov Katz, Idit Matot, Noam Mubada, Reuven Pizov, Aeyal Raz, Gefen Revaz, Igor Reznikof, Nogzar Rigzny, Michael Rudin, Vladimir Rukinglass, Albert Sabatnitzki, Eran Segal, Eric Siton, Mustafa Somri, Riad Tome, Jacob Turban, Nathan Weksler, Dafna Wilner, Yossi Witchelevsky, Alex Zlotnik

**Affiliations:** 1 Faculty of Medicine of the Hebrew University of Jerusalem, Jerusalem, Israel; 2 Braun School of Public Health, Hebrew University of Jerusalem, Jerusalem, Israel; 3 Hospital Administration, Hadassah-Hebrew University Medical Center, Kiryat Hadassah, POB 12000, 91120 Jerusalem, Israel; 4 Department of Anesthesia and Operating Rooms, Rabin Medical Center, Beilinson Hospital, Petach Tikva, Israel; 5 Sackler Faculty of Medicine, Tel Aviv University, Tel Aviv, Israel; 6 Department of Anesthesiology, Critical Care and Pain Medicine, Hadassah-Hebrew University Medical Center, Jerusalem, Israel

**Keywords:** Anesthesiology, Healthcare policy, On-call hours, Physician workforce, Policy implementation

## Abstract

**Background:**

The Israeli physician workforce faces multiple challenges. These include planned policies reducing physician on-call from 26 to 18 h and, from 2026, allowing only graduates of Ministry of Health approved foreign medical schools to take the Israeli licensing examination and an ongoing physician shortage (2019: Israel had 3.19 physicians/1000 persons vs. OECD average of 3.49 physicians/1000 persons). This study examines the potential impact of these planned policies on the Israeli anesthesiology workforce.

**Methods:**

Surveys conducted among 34 public and private Israeli hospital anesthesiology department chairs collected data on their department's number of weekday on-call anesthesiologists and current shortage of anesthesiologists. A subsequent survey collected data on each anesthesiologist in the workforce, including the country where they studied medicine.

**Results:**

Each weekday night there were 114 on-call anesthesiologists; 72 residents and 42 attendings. Using productive work coefficients, this translates to 104 resident and 51 attending anesthesiologists. Furthermore, 21 departments had existing anesthesia workforce shortages totaling 110 anesthesiologists. There were 873 anesthesiologists from non-OECD countries whose medical schools are not accredited by the World Federation for Medical Education, of whom 332 were residents (61.9% of residents). Only 20.1% of anesthesiology residents were Israeli medical school graduates.

**Conclusions:**

Descriptive survey data assessed the immediate and long-term consequences for the healthcare system and anesthesiology workforce of two new Health Ministry policies. Implementing the 18-h policy will immediately remove from the daytime workforce 155 anesthesiologists and who will be unavailable to staff elective surgery operating rooms. This will compound the current national shortage of 110 anesthesiologists. It is unclear how to replace this shortfall since there are no surplus Israeli physicians and very few Israeli graduates choose anesthesiology as a specialty. This situation will be exacerbated after 2026 when graduates of certain foreign medical schools will be unable to enter the medical workforce, further reducing the pool of potential anesthesiology residents. Both policies were promulgated without adequate operational and budgetary planning or fiscal or workforce resources; implementation of the 18-h on-call policy has already been postponed. Therefore, new or updated policies must be accompanied by specific operational plans, budgetary allocations and funds for additional workforce.

## Background

The Israeli physician workforce faces several current and future challenges, especially the promulgation of two new government policies: The initiative to reduce physician on-call shifts from 26 to 18 h and the "Yatziv Reform" which will permit only graduates of Ministry of Health approved foreign medical schools to take the Israeli licensing examination. The former policy reduces the working hours of the Israel physician workforce requiring the employment of additional physicians, while the latter one reduces the number of young physicians entering the workforce. These impending issues are in addition to an ongoing shortage of physicians per capita which, unless remedied, will be worsened by the continuous growth and aging of the population [1].

A recent policy promulgated by the Israeli Ministry of Health is to reduce physician on-call shifts from 26 to 18 h. This policy was championed by the medical residents’ union (Mersham) as a measure to improve physician well-being and improve patient safety and was embraced by both the Health and Finance Ministries. According to the new policy, during weekdays on-call physicians would work from 4:00 pm to 8:00 am, instead of the current 8:00 am to 8:00 am. With the addition of two hours for hand-over between shifts, this policy effectively reduces the on-call consecutive working shift from 26 to 18 h. This policy was scheduled to be implemented in the periphery of Israel in February 2022, a mere 4 months after its promulgation, with nationwide implementation by 2027. The policy has been suspended pending further review. Proponents of the policy argue that it will reduce direct patient injury from fatigue-related human error and may reduce the effects of fatigue-related stress on the physical and mental health of physicians. However, there are also potential drawbacks to the proposal. Not working on the day prior to night call interrupts the residents’ day-time teaching, reduces their clinical exposure, and interferes with continuity of care—all factors which may adversely affect patient safety. However, among the immediate operational problems is that this policy would require that physicians would no longer be available to provide clinical services during the daytime prior to call. However, no provisions were made to assess workforce requirements or for the employment of additional physicians prior to promulgating this policy. As anesthesiology is a specialty with an established chronic workforce shortage it is likely that this new policy will likely have a major impact on elective surgery schedules [2].

The second impending policy initiative is the “Yatziv Reform”. This policy, scheduled to go into effect in 2026, will restrict the Israeli physician licensing examination for foreign medical school graduates to only those who completed their medical training in institutions approved by the Ministry of Health [3]. The reason for this policy initiative is the poor pre-clinical and clinical medical education provided by some medical schools often attended by Israeli citizens studying abroad. The authors of the “Yatziv Reform” noted that graduates of these medical schools often lack the knowledge required to pass the Israeli medical licensing examination and lack the skills required to provide adequate medical care to patients. All medical schools from countries in the OECD will be automatically approved, but only selected medical schools in non-OECD countries will be approved. Approval will be restricted to medical schools accredited by the World Federation for Medical Education and who provide training in accredited hospitals. It has been estimated that this policy will reduce the number of physicians in Israel to 2.9 physicians per 1000 persons by 2035 unless measures are taken to increase the number of medical students in Israeli medical schools and increase the Israeli students studying in approved medical schools abroad [4]. This policy is expected to significantly worsen the workforce shortage in anesthesiology, since both Israeli citizens and foreigners who are graduates from non-approved medical schools in non-OECD countries (particularly in Eastern Europe, Central Asia, Africa and South America) constitute a significant number of anesthesiology residents.

These two policy initiatives, reducing on-call working hours, and restricting medical licenses to certain overseas medical schools, are both well-intentioned and aim to improve patient safety. However, they both require additional physician workforce which is not currently available in Israel. Therefore, both policies have major implications for the ability of the Israeli workforce, in general, and the anesthesia workforce, in particular, to meet clinical needs which, if not addressed, will cause disruption to clinical services. This situation highlights how there can be a disconnect between policies formulated within a government ministry responsible for health of the population and the health care delivery system that must implement the policy. i.e., implementation considerations are unavoidable part of the process, yet in these cases were avoided. Specifically, operational and budgetary planning, including workforce planning, should always occur prior to the promulgation of policy. Therefore, the present study provides an example of the type of analysis that should have been performed during the development of these two policies by examining the effects of these policies on the Israeli anesthesiology workforce. Anesthesiology is a vital medical specialty essential in the performance of surgeries and other medical procedures. However, it is a specialty with disproportionately large numbers of overseas graduates and plagued by chronic workforce shortages, which already limits the number of surgeries and procedures performed in certain hospitals [2].

## Methods

In August 2019 and March 2021, the Israel Society of Anesthesiologists conducted two surveys of anesthesiology workforce among the anesthesiology department chairs in all 34 public and private Israeli hospitals. The hospitals were identified using the Ministry of Health’s website and confirmed using information from the Israel Society of Anesthesiologists’ list of anesthesiology department chairs. These surveys were sent by one of the authors (YG) on behalf of the Israel Society of Anesthesiologists to the chairs via e-mail. Follow-up telephone calls were made (initially by a research assistant and/or YG and, if necessary, by the chair of the Israel Society of Anesthesiologists—SF) to all the chairs who did not initially reply and also to request the completion of incomplete information and to clarify unclear information. Due to the thorough follow-up all surveys were completed. The timeline for each survey was approximately 4 months. As background, Israel has a population of 9.36 million inhabitants (2021), with about 14.2 anesthesiologists per 100,000 population and approximately 550,000 surgeries are performed annually.

In the August 2019 survey was performed in preparation for negotiations for renewal of the contract between the Israel Medical Association and the Israeli Government (these negotiations were postponed due to the intervening Covid-19 pandemic and have yet to begin). The data requested included the number of on-call anesthesiologists (residents and attendings) on weekday evenings/nights (Sunday-Thursday) and during the weekends (Friday and Saturday). In addition, they were also asked if there was a shortage of anesthesiologists needed to provide adequate service in their hospital. If so, they were asked to quantify this deficit by replying how many operating rooms (if any) were cancelled on an average day due to anesthesiology workforce shortfalls, and to state whether this shortfall was due to a lack of suitable applicants for authorized positions or due to a refusal of the hospital administration to approve additional positions (Appendix 1).

The subsequent survey in March 2021, was performed in further preparation for negotiations for renewal of the contract between the Israel Medical Association and the Israeli Government and to gauge the possible effects of the Yatziv reforms. This survey requested detailed information on the composition of the Israeli anesthesiology workforce. Deidentified data were collected about each anesthesiologist, their age, gender, whether they were a resident or attending anesthesiologist and the country where they studied medicine (Appendix 2). This information was used to determine the number and proportion of current anesthesiology residents who are graduates of medical schools from non-OECD countries whose future graduates will not be permitted to take the Israeli licensing examination beginning in 2026.

### Data analysis

The data collected in the two surveys were entered into Excel (Microsoft Inc. Redmond, WA). Excel was also used to perform the calculations and draw the graphs. Anesthesia workforce shortfall data are presented as percentages, as is the proportion of physicians who are graduates of Israeli medical schools, OECD medical schools and non-OECD medical schools.

#### Reducing physician on-call shifts from 26 to 18 h

We used the descriptive data obtained from the August 2019 survey to determine the number of additional new anesthesiologists needed to replace the on-call anesthesiologists who would no longer be available for the morning weekday shift prior to call, following the new 18-h on-call shift policy. The number of vacant spaces that need to be filled is equal to the number of anesthesiologists currently on-call in Israel during weekdays, as determined directly from the August 2019 survey data, multiplied by productive work coefficients. Therefore, the actual number of new full-time hires required to provide this number of replacement anesthesiologists was calculated using locally accepted productive work coefficients: 1.22 for attending anesthesiologists and 1.45 for anesthesiology residents. These productive work coefficients were based on the provisions of the physicians’ union contract between the Israel Medical Association and the Israeli Government in 2011. For residents, the productive work coefficients were also based on the Anesthesiology Residency Curriculum of the Scientific Council of the Israel Medical Association. For both attendings and residents, vacations, radiation vacation days, sick leave, maternity/paternity leave and military reserve duty were contributing factors. For residents, additional contributing factors were the mandatory study leave prior to the two specialty certification examinations, and non-operating room rotations (e.g. ICU, pain management, research, rotations in other clinical departments).

The total shortage following the 18-h shift policy of anesthesiologists was calculated as the number needed to replace those on-call plus the number of unfilled positions and non-authorized positions reported by the department chairs.

#### Yatziv Reform

Using the descriptive data from the March 2021 survey, we determined, the number of physicians (residents and attendings) who are graduates of Israeli medical schools, OECD medical schools and non-OECD medical schools from countries with and without World Federation for Medical Education accreditation. As the Yatziv Reform will not be implemented retroactively on the existing physicians in the workforce, the immediate implications are limited. We calculated the potential impact of the Yatziv Reform on physician recruitment to the workforce after implementation of this policy in 2026, based on the current proportions of physicians from non-approved counties among the different hospitals.

## Results

Data were obtained from all 34 Israeli acute care hospitals (28 public and 6 private hospitals). The April 2019 survey was completed within 2 months of initiation, while the March 2021 was completed within 6 months of initiation.

### Reducing physician on-call shifts from 26 to 18 h

**Fig. 1:**
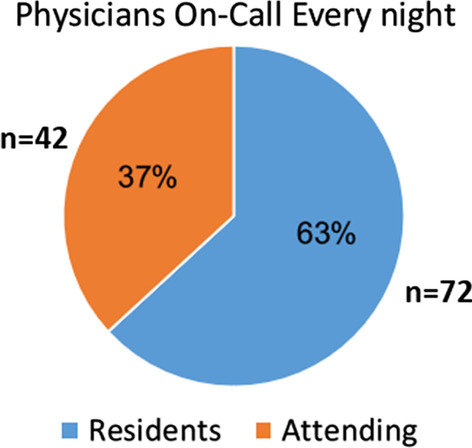
The number of resident and attending physician anesthesiologists on-call each weekday night (Sunday–Thursday)

Based on the combined reports from the department chairs, each weekday night in Israel there are a total of 114 anesthesiologists on call, 72 residents and 42 attendings (Fig. 1). Based on the new 18-h on-call policy, none of these will be available to work on the day prior to call. Using the productive work coefficients, 104 residents and 51 attending anesthesiologists will need to be hired to replace this number of physicians missing from the daily workforce.

**Fig. 2:**
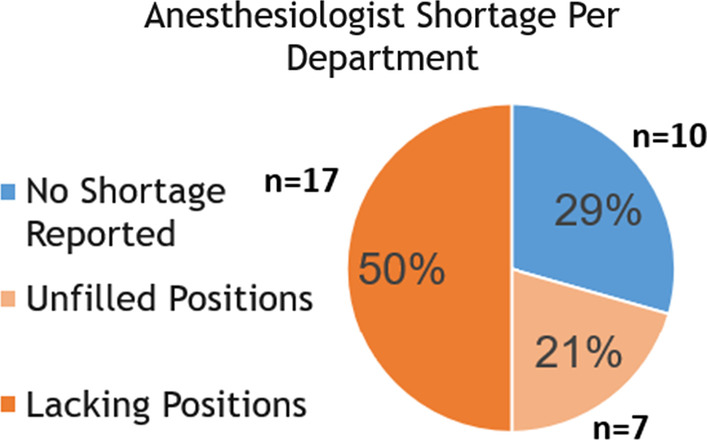
The anesthesiologist shortage per anesthesiology department is shown. Seventy-one percent of departments reported having an anesthesiologist shortfall

In addition, 21 of the 34 departmental chairs reported their departments have an existing anesthesia workforce shortfall totaling 110 anesthesiologists (Fig. 2). When these 110 physicians are added to the 155 needed to replace those on shortened on-call duty a total of 265 additional anesthesiologist are needed.

**Fig. 3:**
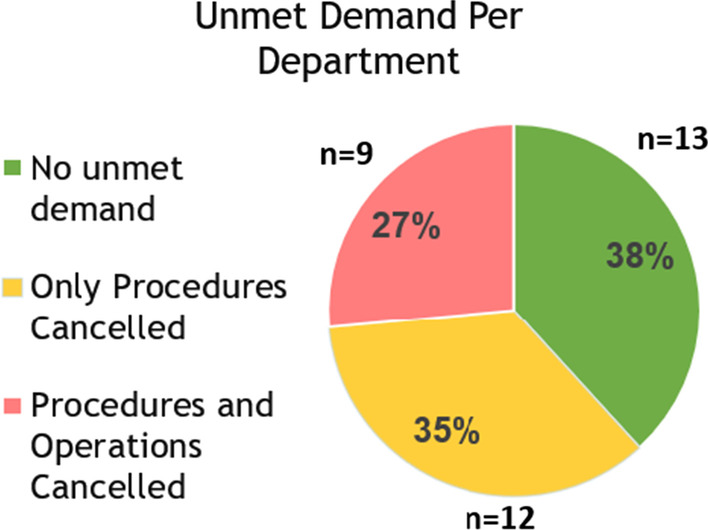
Unmet demand for anesthesiology services was reported by 21 of the 34 anesthesiology departments

We identified a few direct obstacles to recruitment. Of the 24 department chairs reporting current workforce shortages, 7 reported that their current shortfall in workforce was due to a lack of suitable applicants for approved vacancies (18 positions), and 17 stated that this was due to a refusal of the hospital administration to authorize new positions (92 positions). Furthermore, 10 reported that if they were to hire new anesthesiologists, the night-calls for these additional anesthesiologists would come from a fixed pool, effectively reducing the financial compensation of the other attendings in their department (Fig. 3).

### Yatziv reform

The number of anesthesiologists in each department from non-OECD countries was obtained from each department chair. Overall, there were 873 physicians (66.5% of workforce) from non-OECD countries in the Israel anesthesiology workforce. From 2026, most of these physicians would no longer be eligible to join the medical workforce in Israel.

Among the current anesthesiology residents, only 90/447 (20.1%) are Israeli graduates, 178/447 (39.4%) trained in OECD countries and a further 244/447 (54.5%) are from countries where the medical schools are recognized by the World Federation for Medical Education (WFME). Only 25/447 (5.2%) trained in non-OECD countries where the medical schools are not recognized by the WFME (Table 1). However, the situation is more complicated, as the Yatziv report also requires that the individual medical schools are linked to hospitals with international accreditation (e.g. Joint Commission International) and are recognized by the Israeli Ministry of Health. Based on estimates made by the Israeli Ministry of Health, this would prevent the employment of as many as 51% of overseas graduates currently working as anesthesiology residents in Israeli hospitals (Alexey Belinsky, Ministry of Health, Personal Communication).

**Table 1:** Anesthesiology residents in Israel, broken down by countries of medical training

OECD			Non-OECD but WFME recognized			Non-OECD and non-WFME		
Country of medical training	Number of residents per country	Percent of anesthesiology residents in Israel (n = 447)	Country of medical training	Number of residents per country	Percent of anesthesiology residents in Israel (n = 447)	Country of medical training	Number of residents per country	Percent of anesthesiology residents in Israel (n = 447)
Canada	1	0.2	Azerbaijan	1	0.2	Argentina	5	1.1
Colombia	1	0.2	Belarus	5	1.1	Bolivia	1	0.2
Costa Rica	7	1.6	Brazil	2	0.4	Bulgaria	2	0.4
Czech Republic	6	1.3	Egypt	19	4.3	Cameroon	2	0.4
France	2	0.4	Georgia ǂ	17	3.8	Cuba	1	0.2
Germany	14	3.1	Jordan	11	2.5	India	2	0.4
Greece	1	0.2	Kazakhstan	1	0.2	Morocco	1	0.2
Hungary	12	2.7	Moldova ǂ	28	6.3	Pakistan*	2	0.4
Israel	90	20.1	Palestinian Authority	7	1.6	Peru	1	0.2
Italy	23	5.1				Slovenia	1	0.2
Latvia	1	0.2	Romania ǂ	35	7.8	Syria	5	1.1
Lithuania	6	1.3	Russia ǂ	78	17.4	Tunisia*	1	0.2
Mexico	2	0.4	Turkey	2	0.4	Uruguay	1	0.2
Poland	2	0.4	Ukraine ǂ	37	8.3			
Slovakia	7	1.6	Yemen	1	0.2			
Switzerland	1	0.2						
UK	2	0.4						
Total	178	39.4%	Total	244	54.5%	Total	25	5.2%

ǂ Some or all medical schools will not be recognized by the Israel Ministry of Health

* WFME status applied for

## Discussions

These two new policies of the Ministry of Health have positive as well as negative consequences for the Israeli Healthcare System and the anesthesiology workforce, with both immediate and long-term ramifications. The consequences of the 18-h policy will be immediate, since from the first day of implementation, 114 anesthesiologists will be removed from the daytime workforce. In order to avoid disruption to daytime anesthesia services, there is an urgent need to recruit and train an additional 155 anesthesiologists in order to replace them; this number is in addition to the existing shortage of 110 anesthesiologists nationally and the routine need to replace retiring and other anesthesiologists leaving the workforce. This number also does not include the number of anesthesiologists required to cope with projected growth in surgical demand as the general population and particularly the aged population grows in size.

The consequences of the Yatziv reform will be less immediate since the graduates of non-approved medical schools from non-OECD countries already working in the anesthesia workforce will not be affected. However, graduates of non-approved medical schools will not be able to become residents in Israel after 2026. Currently they constitute over half of the anesthesiology resident body. This will likely create an additional overall shortage of anesthesiology residents after 2026. This will further exacerbate the current anesthesiology workforce shortage and impede attempts to meet the added workforce shortfall caused by the 18-h on-call policy.

This analysis is especially important for the Israeli healthcare system since anesthesiologists play a central role in the provision of many vital hospital services by providing anesthesia and sedation in operating rooms and non-operating room locations, such as in the radiology, gastroenterology, cardiology and labor and delivery suites. Additionally, anesthesiologists also staff most intensive care units (ICU) and provide acute and chronic pain management.

The consequences of these two reforms will be promulgated upon an Israeli physician workforce that already suffers from an ongoing physician shortage. Currently, there is an average of 3.49 physicians per 1000 persons in the OECD, while in 2019, Israel had 3.19 physicians per 1000 persons. The number of Israeli physicians is slated to rise to 3.29 per 1000 persons by 2026 and then begin dropping to 3.22 per 1000 persons by 2035 [1]. The predicted drop in the number of physicians per capita is due to the impending retirement of many physicians who arrived in Israel during the wave of immigration from the former Soviet Union in the early 1990's, coupled with an insufficient number of medical school graduates entering the workforce. This drop in the number of physicians per capita will be further aggravated by the annual population growth. The Yatziv Reform threatens to reduce the number of physicians further, to 2.9 physicians per 1000 persons by 2035, unless measures are taken to increase the number of medical students in Israeli medical schools and increase the Israeli students studying in approved medical schools abroad [4].

### Implications for anesthesiologists

Reducing the duration of physician’s consecutive working hours has been a topic of much attention ever since the Libby Zion case in 1984 [5]. At that time, resident physicians had a typical working week of over 110 h with 36-h consecutive on-call duties every second or third night. Since then, call hours have been reduced by legislation, union contracts and other initiatives throughout the developed world. In Israel, maximum weekly working hours are currently 71.5 h and on-call hours are currently limited to 26 h (24 h plus a 2-h overlap period for information exchange with the relief team). This is similar to many European and North American countries [6]. Only, in England, the Czech Republic, Slovenia and Hungry are consecutive on-call hours shorter (13–16 h). The current European Working Time Directive issued by the Council of Europe includes a 48-h workweek while in the United States and Ontario, Canada the workweek has been reduced to 80 h [7]. In the United States, the rules of the Accreditation Council for Graduate Medical Education (ACGME) also include that residents must have one day off in seven and in-house call no more often than every three days, averaged over four weeks. Furthermore, residents must have at least 14 h free of clinical work after 24 h of clinical assignments. There was a period where United States first-year residents had a 16-h limit on consecutive work but that was rolled back after one year (2017) with a return to 26–28-h on-call shifts, as the disruptions occurring with frequent patient hand-offs were affecting patient safety [8]. In many countries with 16-h limits there is an opt-out clause that allows either the resident to choose to work longer hours or for the specialty or hospital to do the same. For example, the ACGME permits Resident Review Committees to grant exceptions to specialty-specific rotations approved by the hospital for up to a maximum of 88 h per week. In the United Kingdom, residents can choose to work 56 h/week instead of 48 h. Germany, Finland, Latvia and the Czech Republic have similar opt-out programs [6]. The opt-out options provide further insight into the difficulties of establishing strict maximum workweeks, including insufficient workforce to provide clinical services and the need of physicians to maintain a reasonable salary.

The rational for reducing physicians’ working hours is based on evidence that long consecutive working hours, as well as night shift work, are detrimental to the physical and mental health of physicians, and lead to medical human errors, endangering patient safety. Long working hours result in chronic sleep deprivation and the resulting fatigue and stress affects job productivity, the incidence of workplace accidents and worker's short and long-term health [9]. Night shift work appears to be associated with increased cardiovascular disease [10, 11]. Sleep deprivation appears to affect mood more than cognitive or motor performance, and, thus, may affect a physician's ability to communicate effectively [12]. Reducing working hours also provides physicians with more time for self-learning which is especially important for residents in training [13].

There has been recognition that tired, inexperienced and minimally supervised residents make more mistakes than those who are well rested, and well supervised [14]. While reducing the duration of the on-call shifts will likely reduce fatigue, it will also reduce experience, and it is not clear what the net benefits will be in terms of patient safety. Whether an 18-h shift results in better and safer patient care than a 24-h shift has yet to be definitively resolved; some studies show an advantage while others do not [15–22]. the downside to the shorter hours, besides the need for more physicians, is more frequent patient handovers. However, many medical communication errors occur during patient handovers [23], especially among high acuity ICU patients [24]. While excessive work hours endanger patient safety, so do poor handovers and inadequate workforce-workload ratios. [24]. In a recent study, harmful medical errors by ICU resident physicians were higher during a schedule that included 16-h shifts compared to a schedule that included shifts of 24 h or more. Importantly, the 16-h schedule also increased physicians’ workload [24]. The latter occurs when the number of on-call physicians is reduced in order to comply with reduced on-call hours mandates. Therefore, to achieve the intended advantages of shorter on-call duration, it is necessary to further increase the physician workforce to avoid reducing the number of on-call physicians. In addition to increasing the workload of the remaining physicians, reducing the number of on-call physicians results in reduced patient services during the on-call hours. The latter is especially important in a service specialty such as anesthesiology where reducing the number of on-call anesthesiologists will result in a decreased ability to provide timely anesthesia for emergency surgeries, epidural analgesia for laboring women and responses to other emergency situations. Therefore, it is imperative that more anesthesiologists be employed to prevent reductions in both elective daytime and urgent night-time services.

Residency training programs aim to provide residents with a comprehensive specialty education and afford sufficient experience and confidence to become full-fledged specialists able to work without supervision. By the end of training, resident physicians should be able to practice independently [25]. There has been much concern that reduced working hours will reduce the clinical exposure of residents, provide less continuity of care, interfere with daytime teaching programs, and reduce exposure to night-time emergency situations [26]. The reduced patient experience is especially of concern in the procedural specialties where residents are expected to assist or perform a prescribed number of cases.

The present study revealed that 37% of the nightly on-call anesthesiologists are attendings, this high proportion is due to the need to have an attending anesthesiologist available at all times to supervise complicated and emergent situations. Other reasons include insufficient number of residents to fill all the on-call positions, and the need to provide adequate salary. Many of these attending anesthesiologists are in their fifties and even sixties. The concerns about the physical toll of 24-h call apply even more to them. Once an adequate workforce source has been secured to allow the 18-h policy to be implemented, it will need to be applied equally to both residents and attendings who perform on-call duties. In summary, the 18-h policy will have substantial effects on the functioning of anesthesiology departments.

### Implications for the Israeli healthcare system

The intention of the two new Ministry of Health policies is to make positive contributions to the healthcare system. Reducing the on-call hours will result in better rested residents and attending anesthesiologists, with the assumption being that they will provide better and safer patient care. This is especially important for anesthesiologists who must remain vigilant while administering anesthesia at all hours and be able to rapidly respond to changes in their patient's condition [27, 28]. This policy would also be expected to reduce stress, improve health and wellness resulting in fewer sick days. Similarly, the policy to restrict the Israeli medical licensing examination for graduates of foreign medical schools to only those who graduated from approved medical schools is intended to improve the quality of physicians providing care in Israel. However, the healthcare system will not be improved in the short term if these reforms lead to critical workforce shortages and cancelled surgeries. The ability to locally recruit, train and retain additional medical students and physicians into the specialty of anesthesiology will require substantial investment. In addition, it is vital that the initiatives already being implemented to increase the total number of Israeli medical school graduates be accelerated and expanded.

Achieving the intended goals of these two policies will involve major disruptions in anesthesia and other healthcare services unless an orderly plan is developed to implement these changes. There is currently an existing shortfall of anesthesiologists in the national workforce which leads to the cancelation or postponement of elective surgeries in 21/34 (61.7%) of hospitals in Israel; predominantly in peripheral hospitals. There are thus insufficient anesthesiologists in the current workforce to be able implement the 18-h on-call policy; particularly in the periphery, which was proposed to be the location for the first wave of implementation. The future reduction in the recruitment of anesthesiology residents from non-approved medical schools will further aggravate this shortfall, especially since many peripheral hospitals rely on graduates of such schools to fill their anesthesiology residency programs, upon which their clinical service depends. In parallel, the demand for anesthesia services is rising due to an expanding and aging population and due to medical advances [29]. Therefore, it is imperative that the healthcare system advance its policies in an orderly and systematic fashion without disturbing the surgical/procedural volume.

### Implications for hospitals

The consequences of the two policies will affect different hospitals differently. At the time of promulgation of the 18-h on-call policy, implementation was scheduled to occur in peripheral hospitals within 4 months, and to be extended to centrally located hospitals only within 5 years. This immediate implementation did not consider the time required to recruit and train new anesthesiology residents. Furthermore, the more-immediate implementation was scheduled for the peripherally located hospitals, which suffer from the worst workforce shortages, while the more gradual implementation was scheduled for the centrally located hospitals which have better workforce staffing.

Similarly, the hospitals that will likely be most affected by the Yatziv reform are located in the periphery. The residency programs in these hospitals overwhelmingly recruit graduates of foreign medical schools, many of which will not be approved by the Israeli Ministry of Health after 2026. By contrast, the anesthesiology residency programs in hospitals located in the center of the country are filled with many Israeli graduates and are likely be less affected by the Yatziv reform.

It is planned that over the next few years the number of Israeli medical students will gradually increase from 800 to 1200 per year plus 130 per year from the conversion of the three programs training foreign medical student to training Israelis. While these numbers will improve the overall numbers of incoming Israeli medical graduates, this does not address the existing discrepancies in distribution. Anesthesiology remains an unpopular career choice among Israeli medical graduates and the periphery remains less popular than the center. Natural competitive market forces make it likely that anesthesiology will attract a relatively small proportion of the additional Israeli medical graduates, and that those choosing anesthesiology will still choose the center over the periphery.

Data available from the Ministry of Health coupled with data collected in our second survey provided an assessment of Israeli medical school graduates choosing anesthesiology for residency. In 2020 (the latest published data) there were 110 new anesthesiology residents (an increase from 84 in 2019). This constituted 6% of the 1835 physicians who received licenses in 2020. Only 22 (20%) of the 110 new residents were graduates of Israeli medical schools which was 2.75% of the 800 medical school graduates. Therefore, the planned increase in the number of Israeli graduates to 1200 or even 1330 would only result in 33 or 37 new anesthesiology residents per year, respectively, instead of the 22 residents currently. This would hardly provide sufficient anesthesiology residents to reduce the shortfall caused by the two policies. However, it is important to note that there has been an overall increase in the number of new anesthesiology residents over the past 10 years, from 36 in 2010 to 110 in 2020. This is due in part to the incentives provided by the 2011 agreement between the Israel Medical Association and the Israeli government. However, half of the current resident are graduates of foreign medical schools that will not be recognized under the Yatziv program. Therefore, it might be necessary to re-introduce the 2011 incentive program and strengthened it substantially in order to attract more graduates of Israeli and approved foreign medical schools, although there would probably still be a workforce shortfall. Another possibility is to combine residency programs in the central portion of the country with those in the periphery such that residents are exposed to medical care in both areas, as in done in many countries. However, this will likely require much coordination between hospitals. Working conditions should also be improved to convince medical students to choose a career in anesthesiology and, especially, to choose residencies in peripheral hospitals. The later could be facilitated by reinstating the 2011 program that provided incentives for choosing residencies in peripheral hospitals. However, it also may be necessary to consider additional ways of providing anesthesiology workforce.

The effects of these two policies on anesthesiology will be felt at the local level, especially in hospitals that today are already suffering from workforce shortages and that are struggling to maintain their anesthesiology workforce. It is important to note that even without these two policies, hospitals are already under pressure to expand their existing anesthesiology workforces to meet the demands for more surgical/procedural services caused by the growing and aging population. This confluence of a pre-existing workforce shortage together with these two new policies make it necessary that urgent measures be instituted to attract significant numbers of medical students to anesthesiology and to the periphery.

### Strengths and limitations

The strength of this study is that it provides information on the effects of two significant policy changes on a pivotal specialty already suffering a workforce shortage. Furthermore, it uses objective data collected from all the private and public hospitals providing a comprehensive picture of the situation facing anesthesiology throughout Israel. The limitation of this study is that uses anesthesiology departments chairs as the predominant source of information and it only provides information on one specialty. However, all specialties will suffer workforce shortages, including the surgical specialties and subspecialties. Therefore, the reduction in daytime elective surgery due to the decrease in on-call hours will not only be due to insufficient numbers of anesthesiologists, but also due to fewer available surgical residents. Therefore, similar studies should be performed among all specialties and subspecialties to ascertain the overall effects of the two Ministry of Health reforms.

## Conclusions and policy recommendations

Major healthcare policy decisions, such as reducing the length of consecutive physician on-call hours, have many unforeseen ramifications. Such policies must be practical and include comprehensive considerations of the policy's short and long-term downstream effects on current and future activities, funding, workforce requirements and performance. The plans must also consider how the policy will affect other stakeholders, in this case the different specialties and hospitals. Furthermore, when a new or updated policy is announced it must be accompanied by fiscal and operational provisions that will facilitate its successful implementation. The present study demonstrates the importance of performing a thorough examination of the fiscal and workforce implications of all proposed policies and arriving at potential solutions prior to promulgation. Without the latter, the ability to properly carry out the provisions of the policy might be jeopardized [30, 31]. This was demonstrated by the current study where, if they were implemented as proposed, these two new Ministry of Health policies would almost certainly result in an insufficient number of anesthesiologists to meet current demand, let alone meet the expected increasing demands of a growing and ageing population. The effects of these policies would be longer waiting times for surgeries and fewer staff available for sedation for painful procedures, labor analgesia, critical care units and pain medicine. These factors would negatively impact both the provision of safe and timely health care and the financial revenue of hospitals and ambulatory surgery centers.

In summary:

1. The present study provides a caution against the promulgation of policies without adequate operational planning or fiscal or workforce resources. Such policies are vulnerable to postponement of implementation and curtailment of the scope of the policy leading to disaffection by the public, politicians and special interest groups who lobbied for the policy at the outset. All of these outcomes occurred during the failed implementation of the 18-h on-call policy.
2. To avoid a similar situation in the future, procedures and regulations should be adopted that mandate that all new or updated policies be accompanied by specific operational plans and budgetary allocations, including the funding for the workforce increases needed to implement the new policy [32].

## Data Availability

Data will be available from the corresponding author by request.

## Appendix 1: Questionnaire—August 2019

The Chairs of the Anesthesiology Departments were asked to complete an Excel spreadsheet that elicited the following information.

1. Total number of on-call anesthesiologists (attendings and residents) on duty all night on weekdays—including Intensive Care Unit, if applicable
2. Number of Attending anesthesiologists on duty all night on weekdays—including Intensive Care Unit, if applicable
3. Total number of on-call anesthesiologists (attendings and residents) on duty all night on weekends—including Intensive Care Unit, if applicable
4. Number of Attending anesthesiologists on duty all night on weekdays—including Intensive Care Unit, if applicable. Does your department have a shortage of anesthesiologists? (yes/no)
  - If yes, how many operating rooms are cancelled on an average day due to anesthesiology workforce shortfalls?
  - If yes, is it due to unfilled positions? (yes/no)
  - If yes, is it due to lack of authorized positions? (yes/no)
5. Does your department have unmet demand for anesthesia services? (yes/no)
  - If yes, are only non-operating room procedures cancelled? (yes/no)
  - If yes, are operating room and non-operating room procedures cancelled? (yes/no)

## Appendix 2: Questionnaire—March 2020

The Chairs of the Anesthesiology Departments were asked to complete an Excel spreadsheet that gathered data on each anesthesiologist in their department with the following headings.

1. Year of birth
2. Sex (male or female)
3. Graduated medical school in which country?
4. Completed or doing an Anesthesiology Residency in which country?
5. Full time employee (yes/no)
6. Attending Anesthesiologist (certified specialist) (yes/no)
7. Anesthesiology Resident (yes/no)
8. Neither an Attending or Resident (non-certified) (yes/no)
